# Integrin-independent support of cancer drug resistance by tetraspanin CD151

**DOI:** 10.1007/s00018-019-03014-7

**Published:** 2019-02-18

**Authors:** Soonyean Hwang, Takayuki Takimoto, Martin E. Hemler

**Affiliations:** 1 Department of Cancer Immunology and Virology, Rm SM-520C, Dana-Farber Cancer Institute, 450 Brookline Ave, Boston, MA 02215 USA; 2 Present Address: Department of Internal Medicine, National Hospital Organization Kinki-Chuo Chest Medical Center 1180 Nagasone-cho, Kita-ku, Sakai, Osaka 591-8555 Japan

**Keywords:** CD151, Integrins, Drug resistance, Apoptosis

## Abstract

Tetraspanin protein CD151 has typically been studied as binding partner and functional regulator of laminin-binding integrins. However, we show here that CD151 supports anti-cancer drug resistance independent of integrins. CD151 ablation sensitized multiple tumor cell types to several anti-cancer drugs (e.g., gefitinib and camptothecin), thus increasing apoptosis, as seen using cleaved caspase-3, cleaved PARP (poly (ADP-ribose) polymerase), annexin V, and propidium iodide staining assays. Drug sensitization due to CD151 ablation is integrin-independent, because, (1) effects occurred in cells when integrins were unengaged with ligand, (2) integrin ablation (α3 and α6 subunits) did not mimic effects of CD151 ablation, (3) the CD151^QRD^ mutant, with diminished integrin association, and CD151^WT^ (unmutated CD151) similarly reconstituted drug protection, and (4) treatment with anti-cancer drugs selectively upregulated intracellular nonintegrin-associated CD151 (NIA-CD151), consistent with its role in drug resistance. Together, these results suggest that upregulated CD151 expression may support not only typical integrin-dependent functions, but also integrin-independent survival of circulating (and possibly metastatic) cancer cells during anti-cancer drug therapy.

**Electronic supplementary material:**

The online version of this article (10.1007/s00018-019-03014-7) contains supplementary material, which is available to authorized users.

## Introduction

CD151, a 30 kD tetraspanin protein family member, is widely expressed in many tissues and cancer cell types [1–3]. CD151 expression is increased in breast, prostate, lung, colon, skin, and other cancers, and elevated CD151 expression correlates with advanced stage and poor prognosis in many of these human cancers [3–5]. CD151 may variably support tumor initiation, promotion, progression, metastasis, angiogenesis, and drug resistance [6–10]. To date, CD151 has mostly been studied as a closely associated cell surface partner and regulator of laminin-binding integrins (e.g., α3β1, α6β1, α6β4, and α7β1) [11–13]. Genetic evidence reinforces the close functional connection between CD151 [14–16] and laminin-binding integrins [17, 18]. However, CD151 also can appear as a nonintegrin-associated molecule, selectively recognized by anti-CD151 mAb that bind to a CD151 epitope(s) only exposed when it is not bound to integrin [19–22]. A correlation between nonintegrin-associated CD151 (NIA-CD151) and diminished prostate cancer patient survival [21] underscores the potential importance of NIA-CD151.

The absence of CD151 sensitized normal mouse skin cells to trastuzumab, camptothecin, DMBA (7,12-dimethylbenz[α]anthracene), and agents targeting Jak2/Tyk2 and STAT3 [6]. These findings hinted at a possible link between CD151 and general drug resistance. Cancer drug resistance, intrinsic or acquired, can arise due to a variety of mechanisms, including tumor cell heterogeneity, drug efflux and metabolism, and drug-induced genetic or epigenetic cellular alterations [23]. Because integrin-mediated cell adhesion contributes to drug resistance [24], it was expected that potential CD151 contributions to drug resistance would also involve integrins. Indeed, in the special case of drugs targeting ErbB2 and focal adhesion kinase (FAK), CD151 supported drug resistance by a mechanism involving laminin and laminin-binding integrins [25]. However, in sharp contrast to those previous integrin-dependent results [25], we here show that nonintegrin-associated CD151 (NIA-CD151) facilitates a more general and previously unreported anti-cancer drug-induced apoptosis.

## Materials and methods

### Cells, antibodies, and other reagents

A431 (epidermoid carcinoma), MDA-MB-231 (breast carcinoma), and A549 (lung carcinoma) cells were from the American Type Culture Collection (ATCC, Manassas, VA, USA) and maintained in Dulbecco’s modified Eagle’s medium with 10% fetal calf serum (Sigma, MO, USA), and 1% penicillin–streptomycin (Invitrogen, Grand Island, NY, USA) at 37 °C in humidified 5% CO_2_. Monoclonal antibodies to CD151 were 11B1 (gift from B. Copeland), TS151r (gift from E. Rubinstein), 5C11, 1A5 (gift from A. Zijlstra), and 14A2H1 (BD Biosciences, San Jose, CA, USA). TS151r and 1A5 selectively recognize the nonintegrin-bound form of CD151 (NIA-CD151) [21, 22]. Antibodies to cleaved caspase-3, PARP, p-mTOR/mTOR, p-AKT/AKT, and p-ERK/ERK were purchased from Cell Signaling Technologies (Danvers, MA, USA). Antibodies to CD9 (MM2/57), integrin β4, glyceraldehyde-3-phosphate dehydrogenase (GAPDH), and β-actin were from EMD Millipore (Billerica, MA, USA). Antibodies to integrins α6, α3, and CD81 and normal mouse IgG were from Santa Cruz Biotechnology (Santa Cruz, CA, USA). Antibodies to CD49f (α6 integrin), phospho-EGFR, and EGFR were from BD Biosciences (Franklin Lakes, NJ, USA). Gefitinib was from Cayman Chemical (Ann Arbor, MI, USA) and DMSO, camptothecin, cisplatin, and phorbol 12-myristate 13-acetate (PMA), were from Sigma-Aldrich. Nifuroxazide was from Dr. David Frank, Dana-Farber Cancer Institute. Lapatinib and U0126 were from LC Laboratories (Woburn, MA, USA). RNAi: siGENOME human CD151 siRNA (D-003637-04), siGENOME human CD9 siRNA (D-017252-04), and siGENOME human CD81 siRNA (D-017257-05) were from Dharmacon, Inc. (Chicago, IL, USA).

### Cell culture and drug treatment

All cells were routinely cultured in 10% fetal calf serum, while adherent to tissue culture plastic. Drug treatment (at times and doses indicated in legends) was typically carried out for cells adherent to tissue culture plastic, in the presence of 10% serum. Exceptions are as follows: in Fig. 2c–e, A431 cells (labeled as “Nonadherent”) were treated with drugs while plated on polyHEMA (which prevented cell adhesion). In Supplemental Fig. 2a, A431 cells (labeled as detached) were drug-treated while suspended in a conical plastic tube, with occasional mild agitation.

### CRISPR/Cas9-mediated gene deletion and reconstitution

Lentiviral particles containing CD151 gRNA and Cas9 were produced as described previously [26]. CD151 gRNA-1 (5′-CACCGGTAAACAGCAGGTACTTG-3′), gRNA-2 (5′-CACCGGCAGTGGGCATCTGGACGC-3′), and gRNA-3 (5′-CACCGGTGGCCAGGTAGGTGCCTG-3′) (IDT DBA, Coralville, USA). Human nonspecific control gRNA [27] was a gift from TC Cheong. CD151^WT^ and CD151^QRD^ mutant template plasmids [13] were gifts from Dr. C.S. Stipp. Silent mutations were introduced into CD151^WT^ and CD151^QRD^ to remove protospacer adjacent motif (PAM) sites and cloned into pLenti CMV Blast empty (w263-1) (Addgene, Cambridge, MA, USA).

### Immunoprecipitation and immunoblotting

Cell lines were lysed in 50 mM HEPES, 150 mM NaCl, 5 mM MgCl_2_, 1% TritonX-100 (1% Brij 97, or 1% Brij 99), 0.5 mM PMSF and protease inhibitor cocktail (Roche Diagnostics, Indianapolis, IN, USA). After 60 min shaking at 4 °C, lysates were collected and centrifuged (14,000 × g, 10 min, 4 °C), and then, immunoprecipitation was carried out prior to immunoblotting. Densitometry quantitation of protein after blotting was performed using Image Quant, version 5.2 software (GE Healthcare, Waukesha, WI, USA) or ImageJ 1.x (National Institutes of Health, Bethesda, MD). Co-immunoprecipitation of CD151 and PKCα was performed as described [28].

### RNA isolation and semi-quantitative real-time PCR

mRNA was isolated from MDA-MB-231 and A431 cells and amplified using OneStep RT-PCR Kit (Qiagen, Venlo, Netherlands). Human CD151 mRNA primers: Fwd 5′-ACTTCATCCTGCTCCTCATCAT-3′, Rev 5′-TCCGTGTTCAGCTGCTGGTA-3′. Human GAPDH mRNA primers: Fwd 5′-GGCATCCTGGGCTACACTGA-3′, Rev 5′-GTGGTCGTTGAGGGCAATG-3′.

### Immunofluorescence and phase contrast microscopy

For confocal analyses, cells on coverslips were fixed with 4% paraformaldehyde at 4 °C for 10 min, permeabilized by 0.1% Triton X-100 in PBS for 5 min, blocked with 2% BSA in PBS (w/v) for 1 h, and immunolabeled with primary, followed by secondary antibody (Alexa 488 or Alexa 594–conjugated IgG) alone or combined (Invitrogen, Carlsbad, CA, USA). Coverslips were mounted with ProLong Gold antifade mounting media containing DAPI. Cells were visualized using Yokogawa spinning disk confocal microscope (Yokogawa, Japan). Phase contrast images were obtained with Nikon Eclipse TE300 inverted microscope analyzed with Spot software (SPOT Imaging Sterling Heights, MI, USA).

### Cell death assay

Apoptosis was determined using cleaved caspase-3 (immunoblotting), Cell Death Detection ELISA kit (Roche Molecular Biochemicals, Mannheim, Germany), and FITC Annexin V Apoptosis Detection Kit 1 (BD Pharmingen, Billerica, MA, USA).

### Flow cytometry

Adherent cells were detached (using trypsin), and then, cell surface expression of CD151 was quantitated by flow cytometry (FACS Calibur, Becton–Dickinson, Bedford, MA, USA, with processing by FlowJo software, Ashland, OR, USA). Annexin V and propidium iodide staining were quantitated by flow cytometry (FACSAria II, with FACSDiva software analysis, BD Biosciences, Franklin Lakes, NJ, USA) with a minimum of 20,000 cells analyzed in each experiment.

### Statistics

**Fig. 1:**
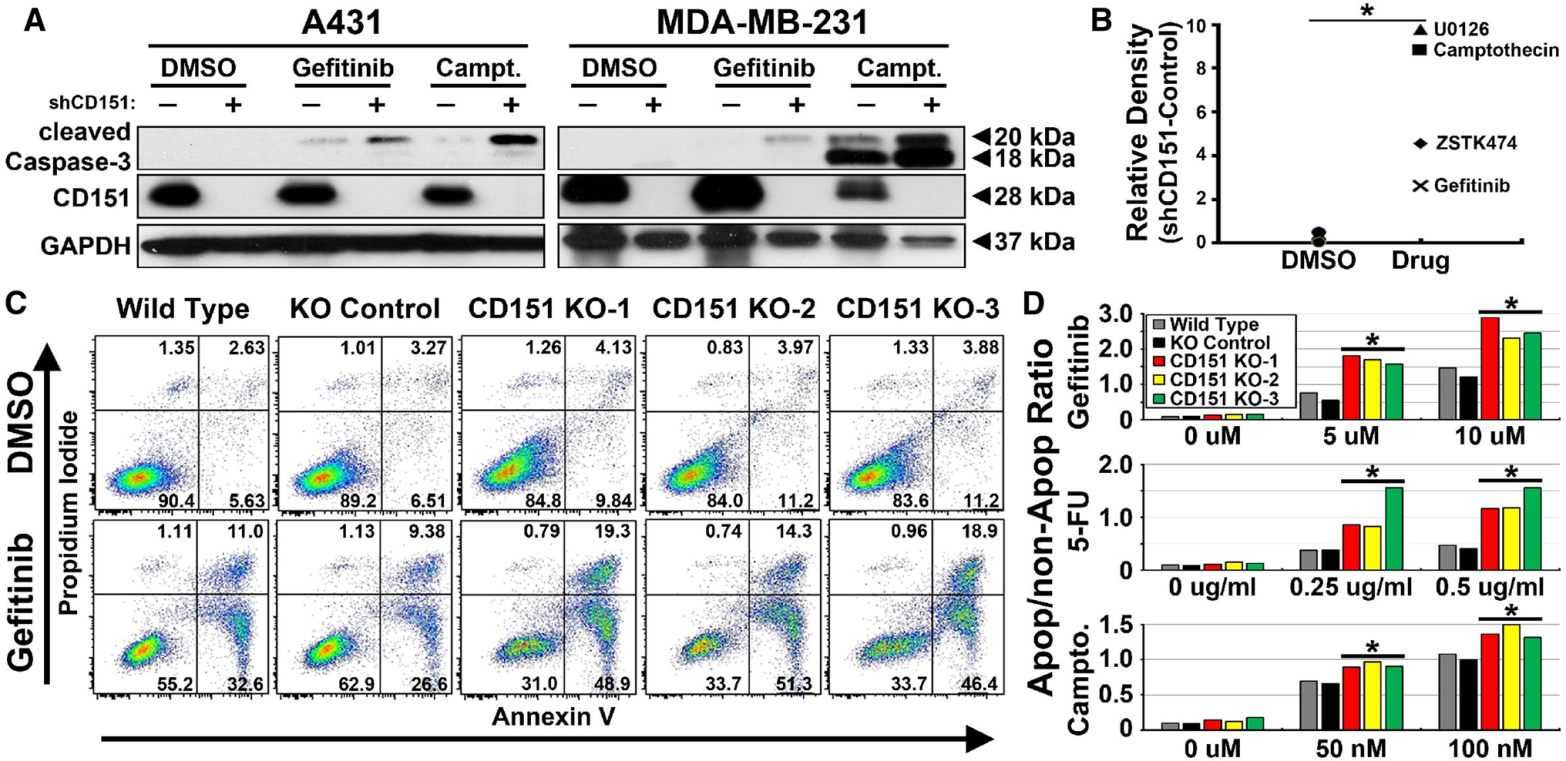
CD151 ablation increases drug-induced apoptosis. **a** A431 and MDA-MB-231 cells ± shRNA-mediated CD151 knockdown were treated for with gefitinib (20 µM, 24 h) or camptothecin (1 µM, 48 h) or DMSO (1:1000, vehicle control). Cell lysates were blotted for cleaved caspase-3, CD151 or GAPDH as indicated. **b** A431 cells, ± CD151 knockdown, were treated with DMSO (1:1000), Gefitinib (10 µM), Camptothecin (1 µM), ZSTK474 (1 µM), and U0126 (10 µM) for 6 h, cell lysates were blotted for cleaved caspase-3, and results were quantitated. *p < 0.05 (comparing four drug treatments with three DMSO treatments). **c** A431 cells with or without CRISPR/Cas9 *CD151* deletion were treated with DMSO or gefitinib (5 µM for 48 h) and then dually stained with annexin V and propidium iodide. **d** A431 cells were treated with gefitinib, 5-fluorouracil, or camptothecin for 48 h. Bars represent ratios of cells positive for annexin V and/or propidium iodide divided by double negative cells (lower left quadrants in panel **c**). *p < 0.05 (comparing three CD151 knockout cell lines with and wild-type and gRNA control cell lines)

**Fig. 2:**
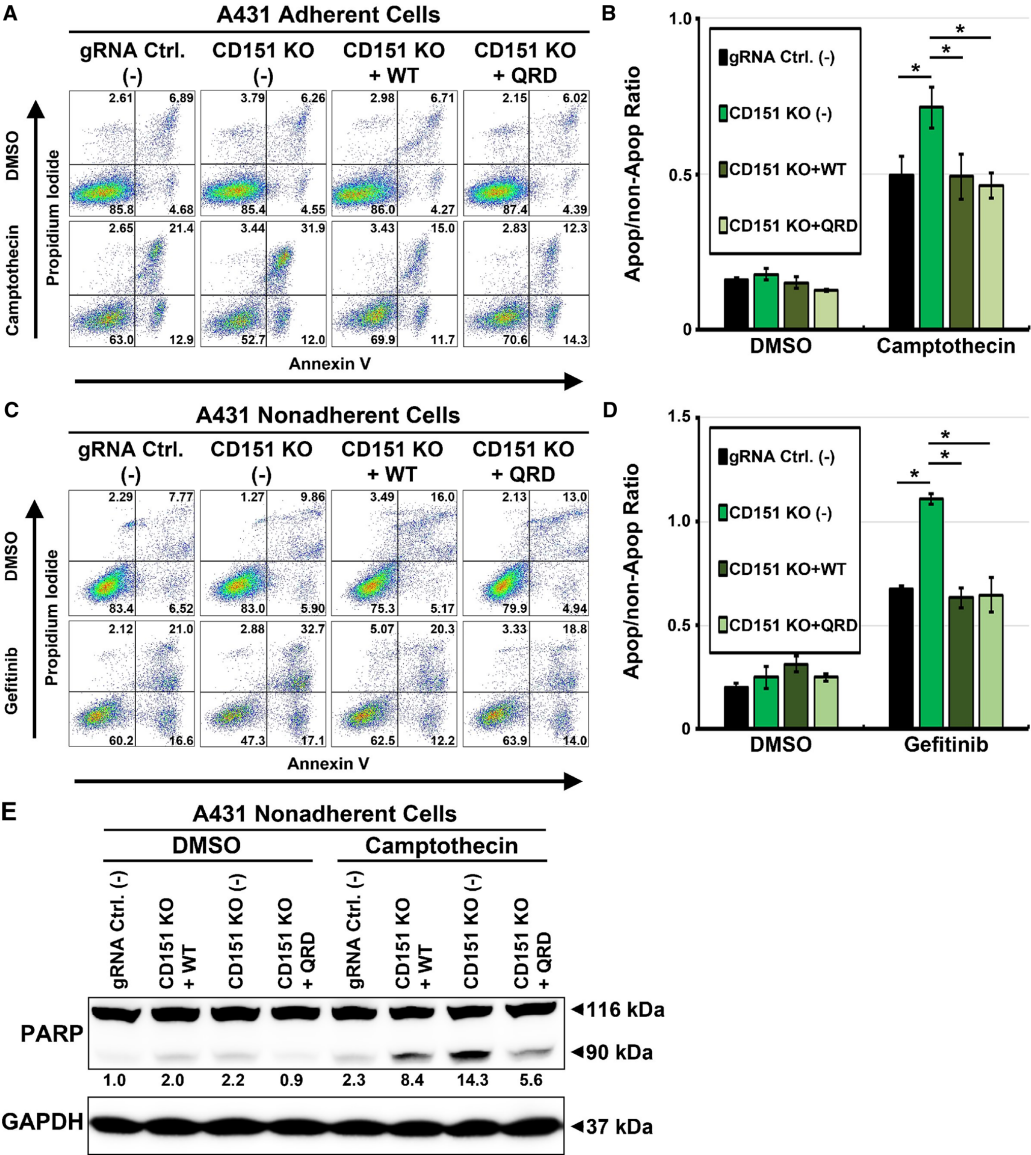
Rescue of anti-cancer drug-induced apoptosis in *CD151* deleted cells. **a** Representative FACS density plots of annexin V and PI staining of adherent cell lines (CD151 knockout, CD151^WT^ and CD151^QRD^ reconstituted) treated with camptothecin (50 nM for 48 h) in adherent conditions. **b** Quantitation of FACS results (*n* = 3; *p < 0.05). **c** Representative FACS density plots of annexin V and PI staining of nonadherent cell lines (CD151 knockout, CD151^WT^ and CD151^QRD^ reconstituted) treated with gefitinib (5 μM for 48 h) on polyHEMA-coated surfaces. **d** Quantitation of FACS analysis (*n* = 3; *p < 0.05). **e** Immunoblot of apoptotic marker cleaved PARP in nonadherent cells treated with Camptothecin on polyHEMA-treated surfaces. Numbers indicate relative density of cleaved PARP normalized to that in DMSO-treated control cells

**Fig. 3:**
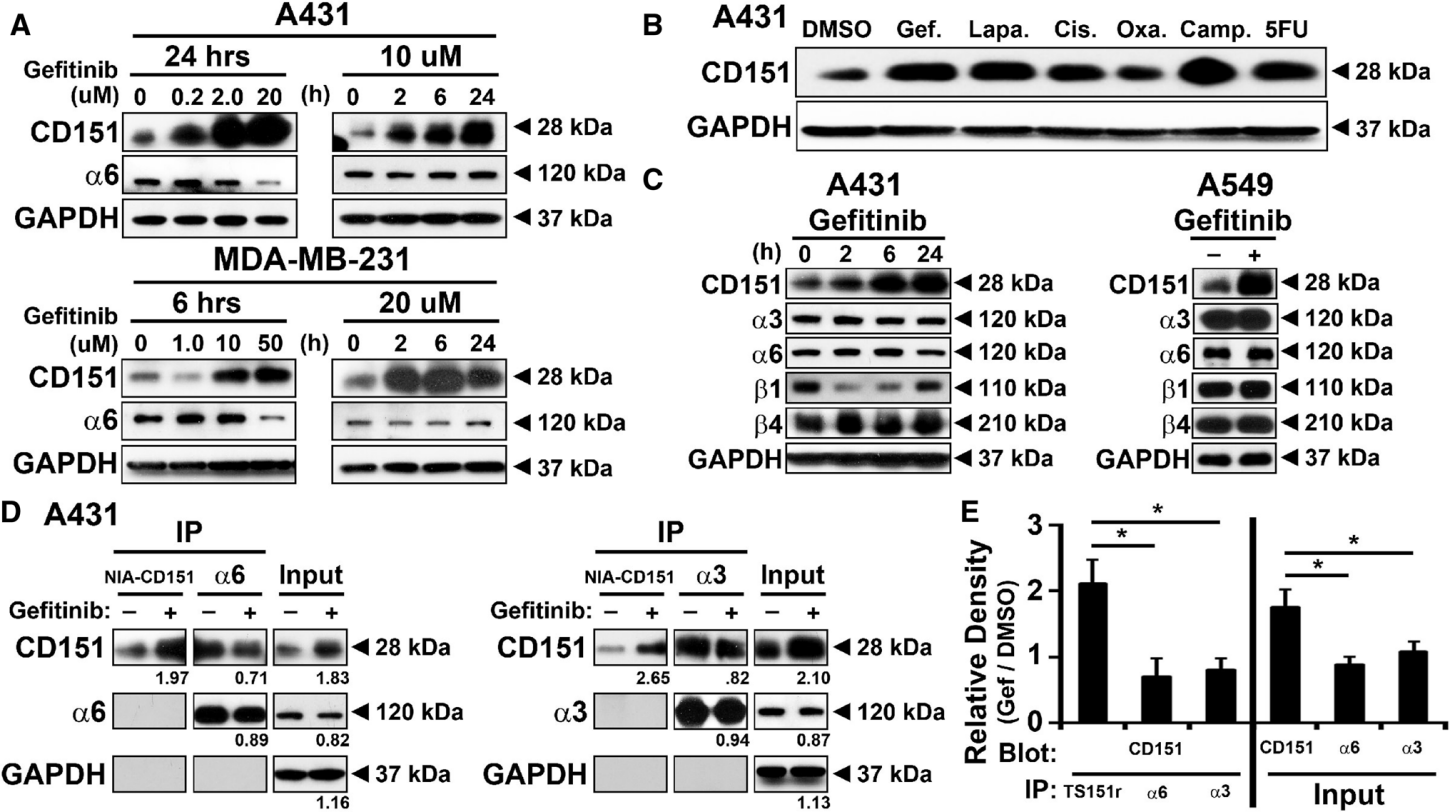
Anti-cancer drug treatment increases nonintegrin-associated CD151 protein levels. **a** A431 and MDA-MB-231 cells were treated with gefitinib for indicated doses and times. Cell lysates were then blotted for CD151, integrin α6 subunit, and GAPDH. **b** A431 cells were treated with the indicated anti-cancer drugs gefitinib (5uM), lapatinib (5 µM), cisplatin (20 µM), oxaliplatine (10 µM), camptothecin (1 µM), and 5-fluorouracil (10 µM) for 25 h, and then, lysates were blotted for CD151 and GAPDH. **c** A431 (for indicated times) and A549 (24 h) cells were treated with gefitinib (20 µM), and then, lysates were blotted for the indicated laminin-binding integrin subunits and GAPDH. D. After treatment with DMSO (1:1000) or gefitinib (20 μM) for 24 h, A431 cells were lysed in 1% Triton X-100. NIA-CD151 (mAb TS151r) and integrins α3 and α6 were immunoprecipitated. Recovered CD151, integrin α6, integrin α3, and GAPDH proteins from immunoprecipitation (lanes 1–4, 7–10) or from input lysate (lanes 5, 6, 11, and 12) were detected by blotting. As expected, anti-NIA-CD151 mAb TS151r did not co-immunoprecipitate integrin subunits (middle panels, lanes 1, 2, 7, 8). Numbers = gefitinib/DMSO treatment ratios from densitometry quantitation. **e** Quantitated ratios are shown for multiple experiments (mean ± SD; *n* = 4; *p < 0.01)

Statistical significance was calculated using two-tailed unpaired Student’s *t* test (Figs. 1b, d, 2b, d, 3e).

## Results

### CD151 attenuates anti-cancer drug-induced apoptosis

CD151 ablation experiments indicate that CD151 protects cancer cells from anti-cancer drugs. Treatment of cancer cell lines (A431 epidermoid carcinoma, MDA-MB-231 breast carcinoma) with gefitinib (EGFR inhibitor) or camptothecin (topoisomerase inhibitor) triggered apoptosis, as evidenced by elevated levels of cleaved caspase-3. The apoptotic response was markedly increased in shRNA-mediated CD151 knockdown cell lines (Fig. 1a; compare lanes 4, 6 with 3, 5 in top panels). A panel of anti-cancer drugs similarly yielded increased apoptosis (i.e., cleaved caspase-3) in CD151 knockdown cells compared to control shRNA cells (Fig. 1b). Consistent with increased apoptosis, gefitinib-treated CD151 knockdown cells also showed a significant decrease in cell growth (not shown). To complement our shRNA knockdown approach, CRISPR/Cas9-mediated deletion of *CD151* was also carried out using three distinct gRNA’s (Supplemental Fig. 1a, b). Consistent with CD151 knockdown results, *CD151* gene deleted cells (CD151-KO) again displayed a marked increase in gefitinib-induced apoptosis, this time as seen by increased Annexin V staining (Fig. 1c; see cell percentage numbers in right panels). In addition, treatment with multiple doses of gefitinib or chemotherapeutic compounds (5-fluorouracil, camptothecin) again significantly increased apoptosis in CD151 deleted cells (Fig. 1d). Consistent with results in Fig. 1a, CD151-KO cells also showed increased drug-induced apoptosis as assessed by blotting for cleaved caspase-3 (not shown).

### CD151 drug protection effects are independent of laminin-binding integrins

CD151 is a regulator of laminin-binding integrins, thus affecting cell motility, morphology, adhesion strengthening, and other functions [3, 19, 29]. However, effects of CD151 ablation on enhanced drug sensitivity remained obvious even when nonadherent cells (i.e., with integrins not engaging ligand) were treated with gefitinib. In fact, enhanced sensitivity due to CD151 ablation was even greater than that seen for adherent cells (Supplemental Fig. 2a). Furthermore, siRNA-mediated knockdown of integrin α3 and α6 subunits (major CD151 binding partners) did not result in increased gefitinib-induced apoptosis (Supplemental Fig. 2b). Together, these results suggest that the effects of CD151 abrogation on anti-cancer drug-induced apoptosis may be independent of CD151 association with integrins.

To further test whether CD151 association with laminin-binding integrins affects the regulation of anti-cancer drug-induced apoptosis, wild-type (CD151^WT^), and CD151 nonintegrin-binding QRD mutant (CD151^QRD^) were reconstituted into *CD151* deleted cells (Supplemental Fig. 3a). Consistent with the published results [19], co-immunoprecipitation of CD151^QRD^, compared to CD151^WT^, shows markedly reduced association with α3 and α6 integrins (Supplemental Fig. 3b). However, despite differences in integrin association, both CD151^WT^ and CD151^QRD^ were similarly able to restore resistance to camptothecin in *CD151* deleted adherent A431 cells (Fig. 2a, b) and resistance to gefitinib in *CD151* deleted nonadherent A431 cells (Fig. 2c, d). Ability of both CD151^WT^ and CD151^QRD^ to restore protection against anti-cancer drug-induced cell death was further verified by diminished appearance of apoptotic marker cleaved PARP (Fig. 2e, lanes 6, 8) in camptothecin treated nonadherent A431 cells. Morphology of A431 cells, grown at either low density or high density, was not noticeably altered due to the deletion of *CD151* or reconstitution with CD151^WT^ or CD151^QRD^ (Supplemental Fig. 3c).

### Anti-cancer drug treatment increases levels of nonintegrin-associated CD151

In various cancer cell lines (A431, MDA-MB-231, and A549 lung carcinoma) treated with gefitinib, we observed an increase in total CD151 protein levels in a dose- and time-dependent manner (Figs. 3a, c, Supplemental Fig. 4a). An increase in CD151 was similarly observed in A431 cells in response to a panel of additional anti-cancer drugs (Fig. 3b). Notably, this increase in CD151 was not accompanied by consistent increases in integrins (α3β1, α6β1, and α6β4) that typically associate with CD151 (Figs. 3a, c, Supplemental Fig. 4a). To test whether anti-cancer drugs selectively induced increases in integrin-associated or nonintegrin-associated CD151, immunoprecipitation experiments were performed. Upon gefitinib treatment, levels of nonintegrin-associated CD151 (NIA-CD151; selectively immunoprecipitated using mAb TS151r) were increased by 1.97-fold (Fig. 3d, left panel) or by 2.65-fold (right panel). By contrast, CD151 co-immunoprecipitated with integrin α3 (right panel) or α6 subunits (left panel) did not increase. A summary of results from multiple experiments confirmed that NIA-CD151 increased by > twofold, whereas integrin-associated CD151 did not increase (Fig. 3e, left). Consistent with the results in Fig. 3a, b, total input CD151 was increased in Fig. 3d (by 1.83- and 2.10-fold) and in Fig. 3e (by ~ 1.8-fold), but not quite to the same extent as the increase in NIA-CD151. In another experiment (Supplemental Fig. 4b), A431 cells either expressed endogenous CD151 (WT) or were deleted for *CD151* and reconstituted with either flag-tagged WT CD151 (WT-F) or flag-tagged QRD CD151 (QRD-F), each under the regulation of a nonendogenous CMV promoter. Again, gefitinib treatment increased CD151 levels, as seen by immunoblotting using three different antibodies (Supplemental Fig. 4b). Increased CD151 was most obvious for mAb 1A5 (recognizing NIA-CD151; top panel) and less obvious for antibodies (mAb 11B1 and anti-FLAG) that recognize total CD151 (next two panels). CD151 protein levels increased in response to gefitinib treatment Supplemental Fig. 4c, but in the same experiments, CD151 mRNA levels did not increase (Supplemental Fig. 4d). These results indicate that the drug-induced increase in CD151 protein does not involve endogenous promotors or enhancers, and does not require integrin association. In drug-treated cells, subcellular distribution of NIA-CD151 (detected using mAb TS151r) showed minimal overlap with integrin α6 (Supplemental Fig. 4e) or integrin α3 (not shown), consistent with NIA-CD151 having a role independent from integrins.

**Fig. 4:**
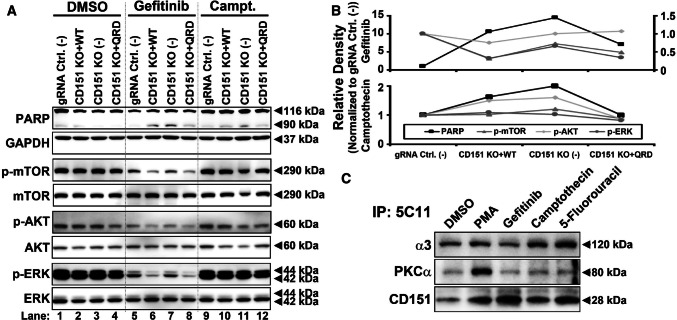
Effects of CD151 changes on pro-survival signaling pathways. **a** A431 cells with *CD151* deleted and/or reconstituted, growing on polyHEMA-coated surfaces, were treated with DMSO, Gefitinib (10 μM, 6 h) or camptothecin (1 μM, 6 h). Cell lysates were then immunoblotted for phospho- and total mTOR, AKTh and ERK, as well as apoptosis marker cleaved PARP and GAPDH. **b** Quantitation relative protein levels are shown for cleaved PARP (left axis), p-mTOR (right axis), p-AKT (right axis), and p-ERK (right axis) normalized to control cell line. **c** A431 cells were treated with control PMA, gefitinib (20 µM), camptothecin (1 µM), and 5-fluorouracil (10 µM) for 8 h. CD151 was immunoprecipitated (using mAb 5C11), and then, levels of recovered CD151 and co-immunoprecipitated PKCα and integrin α3 were assessed by immunoblotting

Although levels of total CD151 and NIA-CD151 were markedly increased upon gefitinib treatment (Supplemental Fig. 4a–c), these increases were not seen at the cell surface on any of the cell lines (A431, MDA-MB-231, and A549) when analyzed for either total CD151 (mAb 11B1 and 5C11) or NIA-CD151 (mAb TS151r) (Supplemental Fig. 5). In contrast, levels of intracellular NIA-CD151 (detected using mAb TS151r) were markedly increased in MDA-MB-231 cells treated with gefitinib (Supplemental Fig. 5b). Together, these results point to selective upregulation of intracellular NIA-CD151 in response to anti-cancer agents.

### CD151 acts as an inhibitor of apoptosis in response to anti-cancer drugs

CD151 itself has not been shown to act as a signaling protein. However, it is implicated in regulating spatial and temporal recruitment of signaling proteins such as conventional PKCs and PI4K [28, 30], which can support pro-survival signaling pathways of cancer cells [31, 32]. Hence, we expected that effects of CD151 deletion and CD151 reconstitution on apoptosis might be opposite to effects on survival, as assessed by phosphorylation of key pro-survival pathway mediators (AKT, ERK, and mTOR). However, no inverse correlation was observed. As indicated in Fig. 4a, the apoptotic marker cleaved PARP was substantially elevated in drug-treated cells lacking CD151 (lanes 7, 11), compared to drug-treated control cells (lanes 5, 9). However, AKT, ERK and mTOR phosphorylations were not correspondingly decreased (lanes 7, 11 compared to 5, 9). Furthermore, in CD151 reconstituted cells (lanes 6, 8, 10, 12), levels of the apoptotic marker were partially diminished (compared to lanes 7, 11), but AKT, ERK, and mTOR phosphorylations were not correspondingly increased. Figure 4b shows densitometric quantitation of results in Fig. 4a.

Induced association of cPKC with CD151 appears to play a key role in cellular growth/survival responses to agents such as EGF and the phorbol ester PMA [6]. However, association of PKCα with CD151 was not altered in cells treated with anti-cancer drugs compared to DMSO (Fig. 4c). In a positive control experiment, PMA did induce an increase in CD151-PKCα association (Fig. 4c, lane 2).

## Discussion

Targeted ablation of tetraspanin protein CD151 sensitized tumor cells to a variety of anti-cancer drugs. Results were seen in multiple tumor cell lines, in which CD151 was either knocked down (using shRNA/RNAi) or knocked out (for the first time using CRISPR/Cas9). Increased apoptosis was indicated by increased levels of cleaved caspase-3, cleaved PARP, annexin V staining, and propidium iodide staining. The increased apoptotic response was associated with reduced growth of CD151-ablated, drug-treated cancer cells.

The previous studies of CD151 have emphasized its close physical association with laminin-binding integrins, and its role in regulating integrin-dependent functions such as cell migration, morphology, and adhesion strengthening [12, 19, 22, 29, 33]. Furthermore, CD151-integrin complexes seemed to be involved in protecting cancer cells in the special case of drugs targeting ErbB2 [25]. Hence, it was unexpected that contributions of CD151 to a more general type of drug resistance would be independent of integrins. Integrin independence is supported by four different types of results. First, drug-sensitizing effects of CD151 ablation were readily observed in nonadherent cells (in which integrins are not engaged). Additional studies using cells on polyHEMA-coated surfaces further indicate that lack of cell–matrix adhesion does not impair drug-sensitizing effects of CD151 ablation. Second, ablation of integrin α3 and α6 subunits did not mimic drug-sensitizing effects of CD151 ablation. Third, the CD151^QRD^ mutant functioned comparably to CD151^WT^ in terms of reconstituting drug protection. As seen previously [19], and confirmed in Supplemental Fig. 3b, CD151^QRD^ has markedly diminished integrin association properties, due to mutation of a CD151 epitope involved in association with α3β1, α6β1, α6β4, and α7β1 [12, 19]. Fourth, anti-cancer drugs failed to induce upregulation of integrin-associated CD151. Rather, they selectively induced upregulation of NIA-CD151, and NIA-CD151-QRD mutant, consistent with the proposed integrin-independent role of NIA-CD151 in drug resistance. Results were mostly obtained using the epidermoid carcinoma A431 cell line. Results obtained using the other cell lines (e.g., breast carcinoma MDA-MB-231 and lung carcinoma A549; representing other carcinoma types) illustrate the generality of our findings, applicable to cells that either do (A431) or do not (MDA-MB-231, A549) produce excess laminin.

Drug-induced selective upregulation of NIA-CD151 does not diminish the pool of integrin-associated CD151 and does not occur on the cell surface. In addition, upregulation still occurs when using a nonendogenous CMV promoter, and is not seen at the mRNA level, thus pointing to a post-transcriptional regulation mechanism. We suspect that enhanced NIA-CD151 expression results from diminished protein degradation, but this remains to be clarified.

**Fig. 5:**
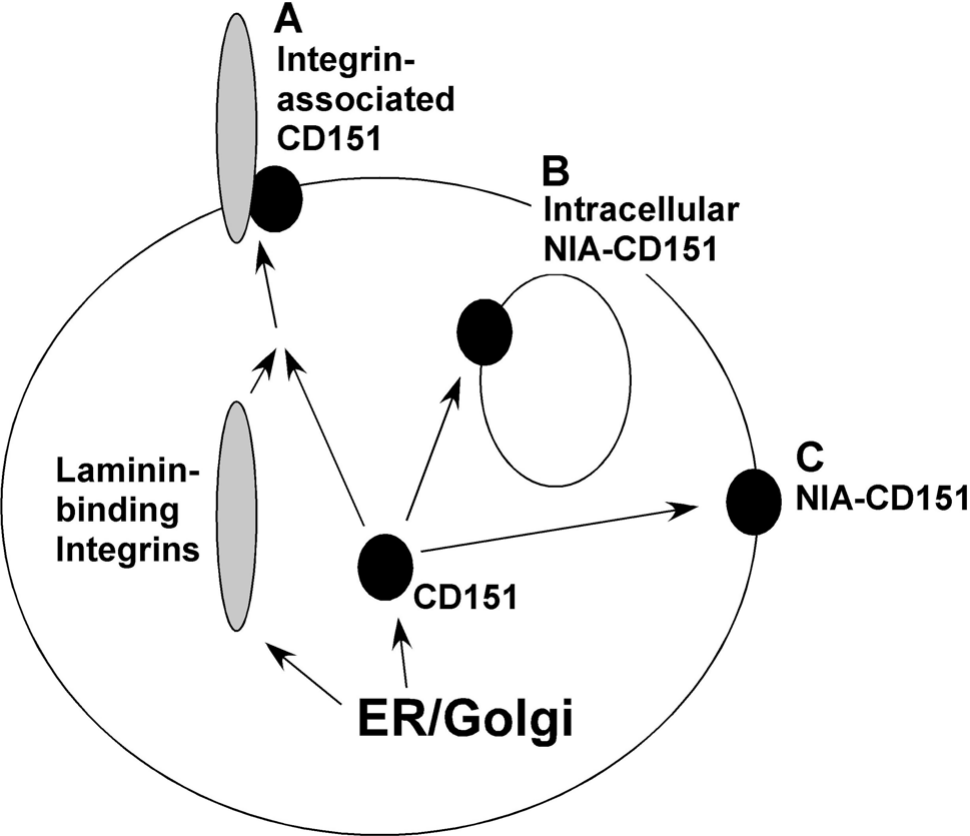
Schematic diagram of CD151 subpopulations. **a** During biosynthesis, a subset of CD151 forms complexes with laminin-binding integrins (e.g., α3β1, α6β1, and α6β4), which then appear on the cell surface. **b** A subset of nonintegrin-associated “NIA”-CD151, localized intracellularly, is upregulated in response to anti-cancer drugs and contributes to drug resistance. **c** NIA-CD151 also can appear at the cell surface. This population of NIA-CD151 is not upregulated by anti-cancer drugs, but may help to regulate cell migration [10]

Together, our results emphasize the existence of at least three distinct populations of CD151 (Fig. 5). (a) Integrin-associated CD151 supports adhesion strengthening, migration and invasion and contributes to multiple stages of carcinogenesis [6–10, 34]. In addition, CD151-integrin complexes have been linked to tumor cell sensitivity to agents targeting ErbB2 [25]. (b) By contrast, intracellular NIA-CD151 is selectively upregulated in response to a variety of anti-cancer agents and contributes to drug resistance. (c) The population of NIA-CD151 that appears variably on the cell surface is not upregulated in response to anti-cancer agents and its functional role is not well defined. However, it was shown that mAb-induced clustering of NIA-CD151 (which is presumably on the cell surface) could inhibit tumor cell metastasis and migration, by a mechanism involving enhanced cell adhesion and perhaps also PKCα [10, 21].

NIA-CD151 expression was previously correlated with prostate cancer progression and diminished patient survival [21]. We speculate that this diminished patient survival could at least partly involve elevated NIA-CD151 levels associated with increased drug resistance. CD151 has previously been considered as a cancer target, due to its contributions to multiple stages of carcinogenesis [2]. Our new results, together with prior results linking NIA-CD151 with patient survival [21], now suggest that it may be therapeutically beneficial to selectively target protein epitopes preferentially exposed in NIA-CD151.

How does NIA-CD151 contribute to drug resistance? The conventional PKC isoforms (e.g., PKCα) can associate with CD151 [6, 28], may be needed for NIA-CD151 regulation of cell migration [21], and can play a role in cancer drug resistance [35]. However, we saw no change in CD151–PKCα association upon drug treatment. Both PKCα [31] and another CD151-associated signaling molecule (PI4K [32]) have been linked to cancer cell survival pathways. However, levels of key intermediates of cancer pro-survival pathways (p-mTOR, p-AKT, and p-ERK) were neither diminished when apoptosis was increased (e.g., in CD151-KO cells), nor elevated when apoptosis was reduced (e.g., in CD151 reconstituted cells). Hence, CD151’s role in attenuating anti-cancer drug-induced apoptosis may be independent of activation of pro-survival signaling. Our results suggest that NIA-CD151 can inhibit drug-induced apoptosis, but a more specific mechanism remains to be elucidated.

In conclusion, our novel and unexpected results now focus attention on a previously understudied subpopulation of CD151 that is nonintegrin-associated and appears to be intracellular. By a combination of nonadherent culture conditions, colocalization, correlation, co-immunoprecipitation, integrin ablation, CD151 ablation, CD151 mutation, and reconstitution experiments, CD151 was shown to inhibit drug-induced apoptosis in an integrin-independent manner.

### Electronic supplementary material

Below is the link to the electronic supplementary material.

Supplementary material 1 (DOCX 13 kb)

Supplementary material 2 (JPEG 117 kb)

Supplementary material 3 (JPEG 147 kb)

Supplementary material 4 (JPEG 295 kb)

Supplementary material 5 (JPEG 311 kb)

Supplementary material 6 (JPEG 113 kb)
