# Supplementary material for: Integrin-independent support of cancer drug resistance by tetraspanin CD151
Source: Cell Mol Life Sci. 2019 Feb 18;76(8):1595–604. doi: 10.1007/s00018-019-03014-7 (PMC6439156; doi:10.1007/s00018-019-03014-7)
Supplement: Supplementary file 1 — Supplementary material 1 (DOCX 13 kb) [file 18_2019_3014_MOESM1_ESM.docx]

**Legends for Supplemental Figures**

**Supplemental Figure 1. CRISPR/Cas9 mediated *CD151* deletion.** A. FACS analysis of surface CD151 expression in control and CD151 knockout cells. B. Immunoblot of CD151 in control and CD151 knockout cells.

**Supplemental Figure 2. Effects of cell adhesion, CD151 and integrin subunits on anti-cancer drug induced apoptosis.** A. A431 cells, ± CD151 knockdown, were treated ± gefitinib (10 µM for 6 hours) while cultured in adherent or non-adherent conditions. Cell lysates were then immunoblotted for cleaved caspase-3, CD151, and GAPDH. B. Adherent A431 cells, ± siRNA-mediated ablation of both α3 and α6, were treated with DMSO or gefitinib (20 µM, 8 hours) and then lysates were immunoblotted for apoptotic markers PARP and cleaved caspase-3, as well as α3, α6, and GAPDH.

**Supplemental Figure 3. Reconstitution of CD151 knockout cells with CD151^WT^ and CD151^QRD^.** A. FACS analysis of surface CD151 expression after sorting of control and reconstituted cells. B. Immunoblot of α3 and α6 integrin subunits co-immunoprecipitated with CD151 (CD151^WT^ or CD151^QRD^) using anti-FLAG antibody. C. Representative bright field microscopic images of CRISPR/Cas9 modified and/or reconstituted A431 cells. Scale bar = 50 µm.

**Supplemental Figure 4. Selective increase in both endogenous and reconstituted CD151 following drug treatment.** A. After A549 cells were treated with gefitinib (20 µM) for indicated times, cell lysates were immunoblotted for CD151, α6, α3 and GAPDH. B. Wild type and reconstituted A431cells were treated ± gefitinib (20 µM, 24 hours) and then lysates were immunoblotted for CD151 using mAb 1A5, mAb 11B1 or anti-FLAG. Note that for CD151^QRD^, the 1A5 epitope is lost and the 11B1 epitope is diminished. C, D. After gefitinib treatment of MDA-MB-231 (20 μM, 24 hours) and A431 cells (10 μM, 24 hours), CD151 and GAPDH were immunoblotted (C) and mRNA levels were quantitated by RT-PCR (D). E. MDA-MB-231 cells were treated with gefitinib (20 μM) for 24 h and then fluorescence was imaged by confocal microscopy. Green, CD151; Red, integrin α6; Blue, DAPI-labeled nucleus. Scale = 10 μm.

**Supplemental Figure 5. Drug treatment increases intracellular, but not cell surface NIA-CD151.** A. Cells were treated for 24 h with DMSO (1:1000) or gefitinib (20 µM, left A431, MDA-MB-231; 5 μM, right A431; 28 μM gefitinib, A549 cells). Mean fluorescence intensity (MFI) of cell surface CD151 was determined by flow cytometry, using indicated anti-CD151 mAb. B. MDA-MB-231 cells were treated with DMSO (1:1000) or gefitinib (20 μM) for 24 h, and then after permeabilization, fluorescence labeling of NIA-CD151 (using mAb TS151r) was imaged by confocal microscopy. Scale bar = 10 μm.
