# Supplementary figures and images for: Integrin-independent support of cancer drug resistance by tetraspanin CD151
Source: Cell Mol Life Sci. 2019 Feb 18;76(8):1595–604. doi: 10.1007/s00018-019-03014-7 (PMC6439156; doi:10.1007/s00018-019-03014-7)

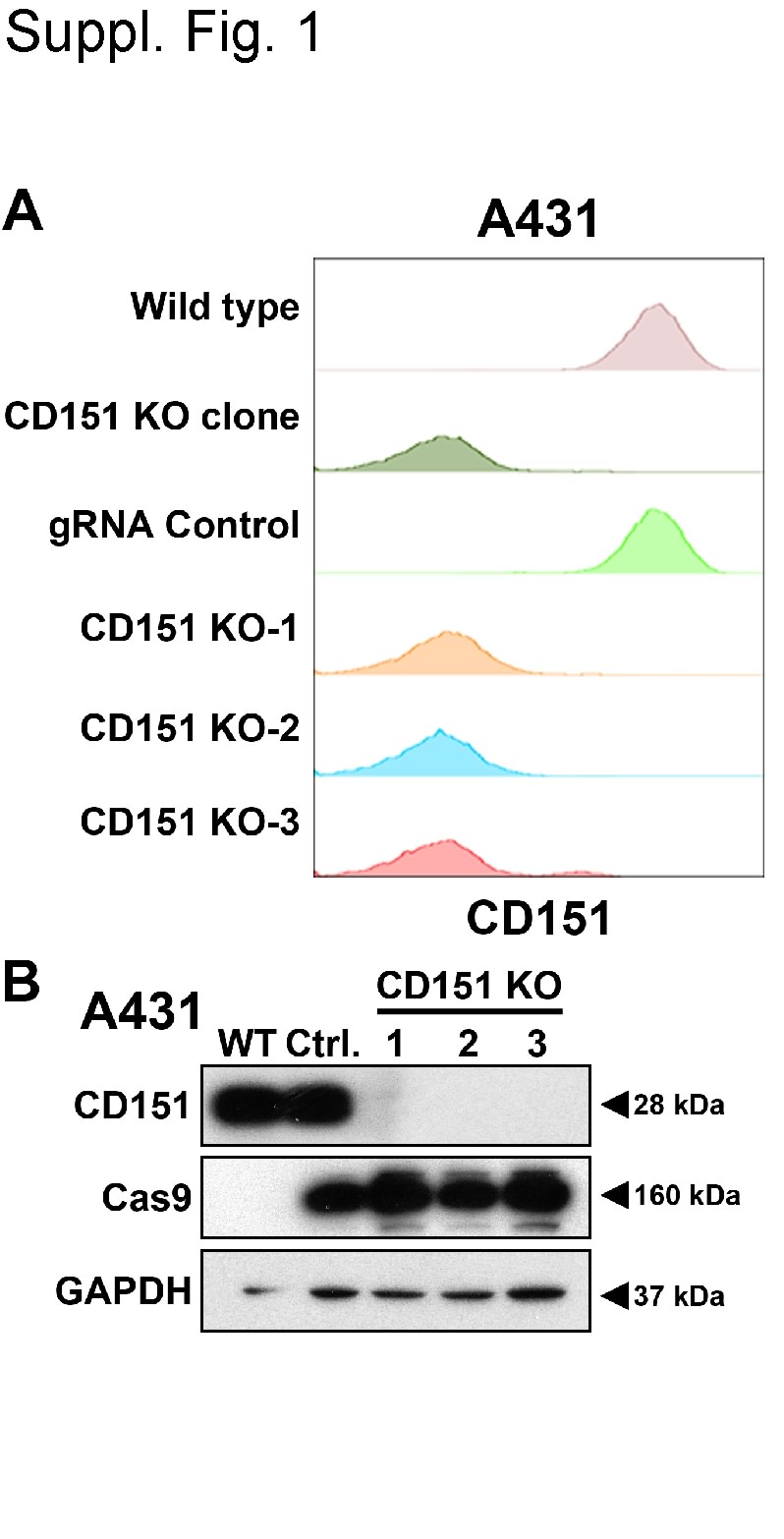

Supplement: Supplementary file 2 — Supplementary material 2 (JPEG 117 kb) [file 18_2019_3014_MOESM2_ESM.jpg]

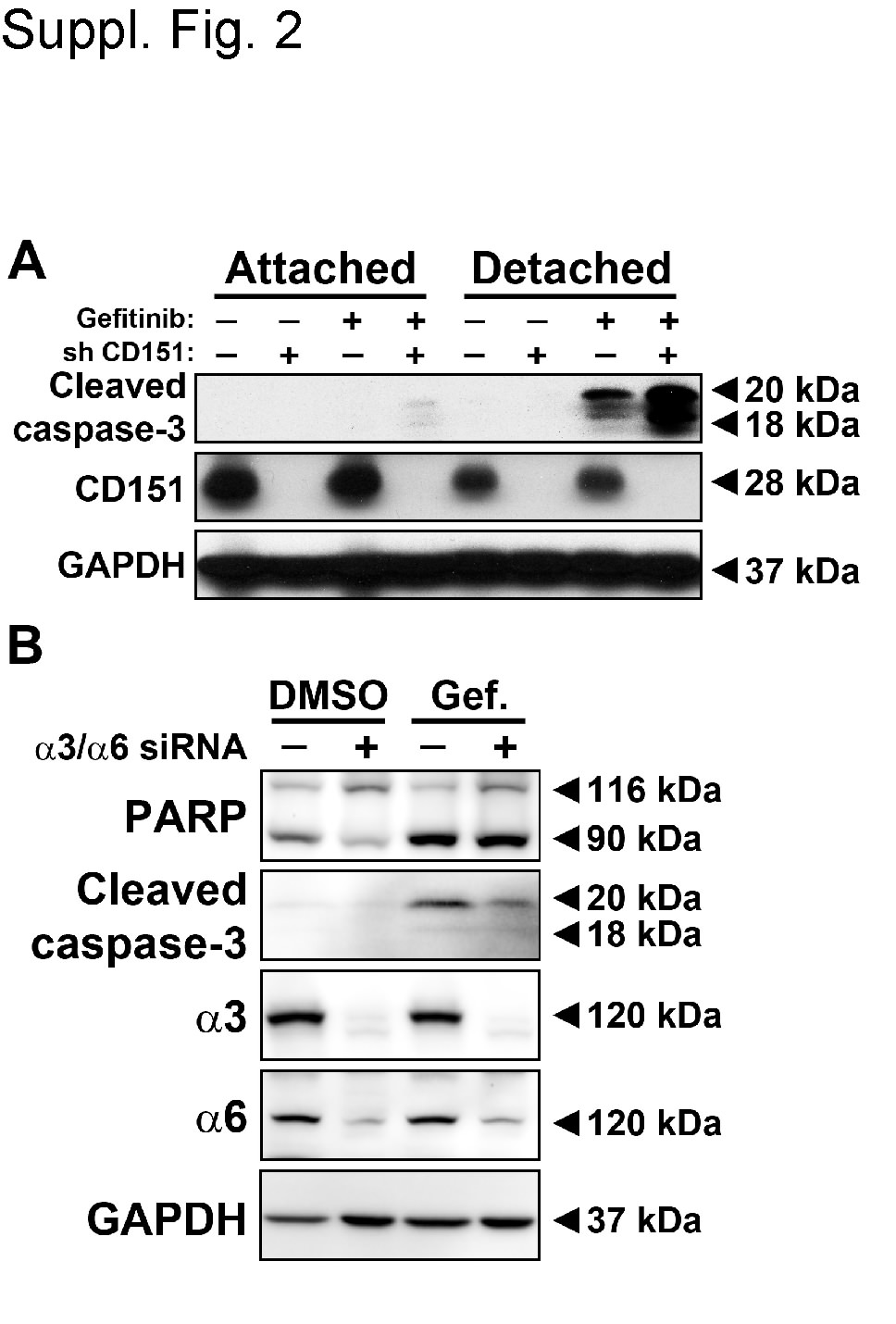

Supplement: Supplementary file 3 — Supplementary material 3 (JPEG 147 kb) [file 18_2019_3014_MOESM3_ESM.jpg]

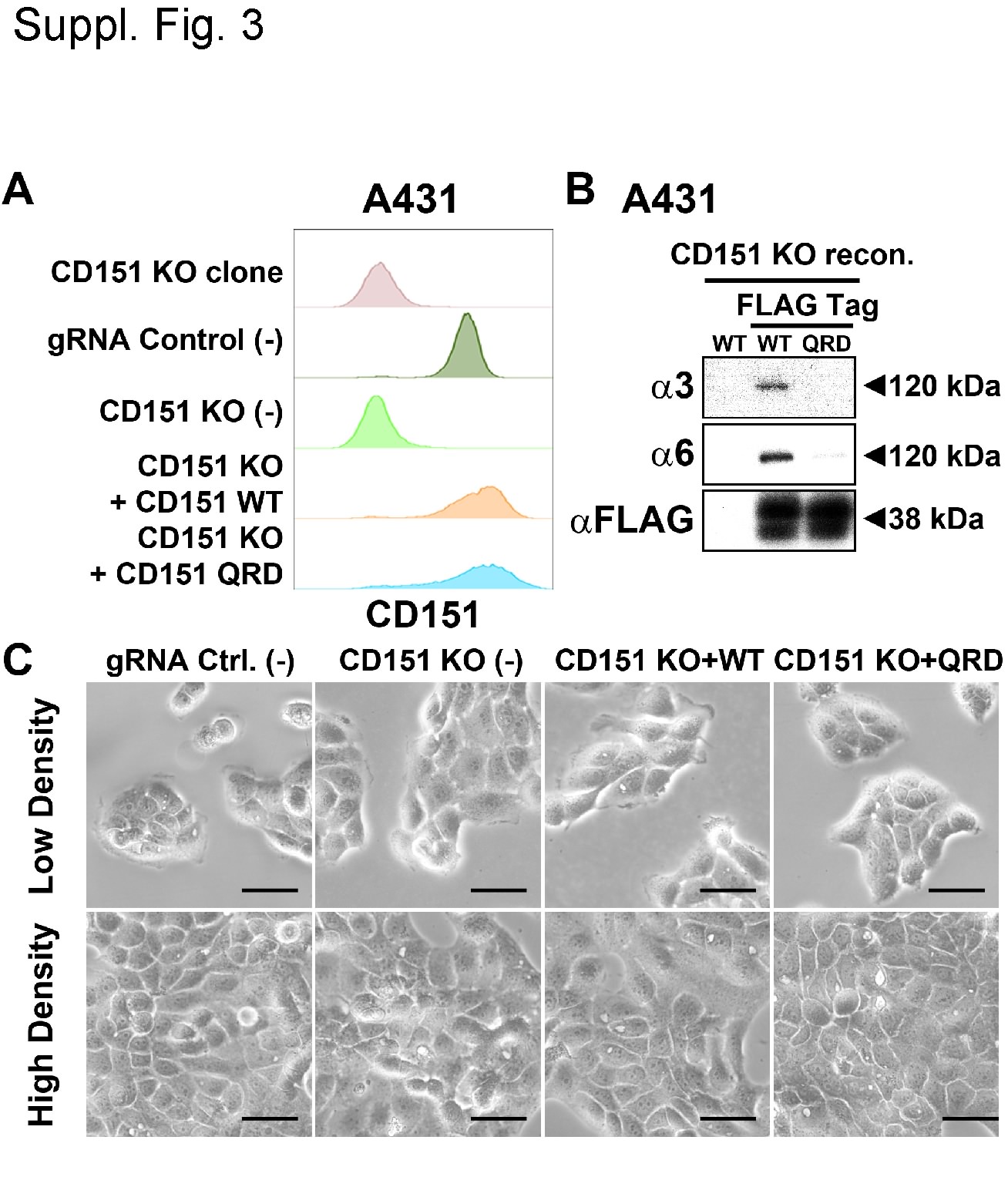

Supplement: Supplementary file 4 — Supplementary material 4 (JPEG 295 kb) [file 18_2019_3014_MOESM4_ESM.jpg]

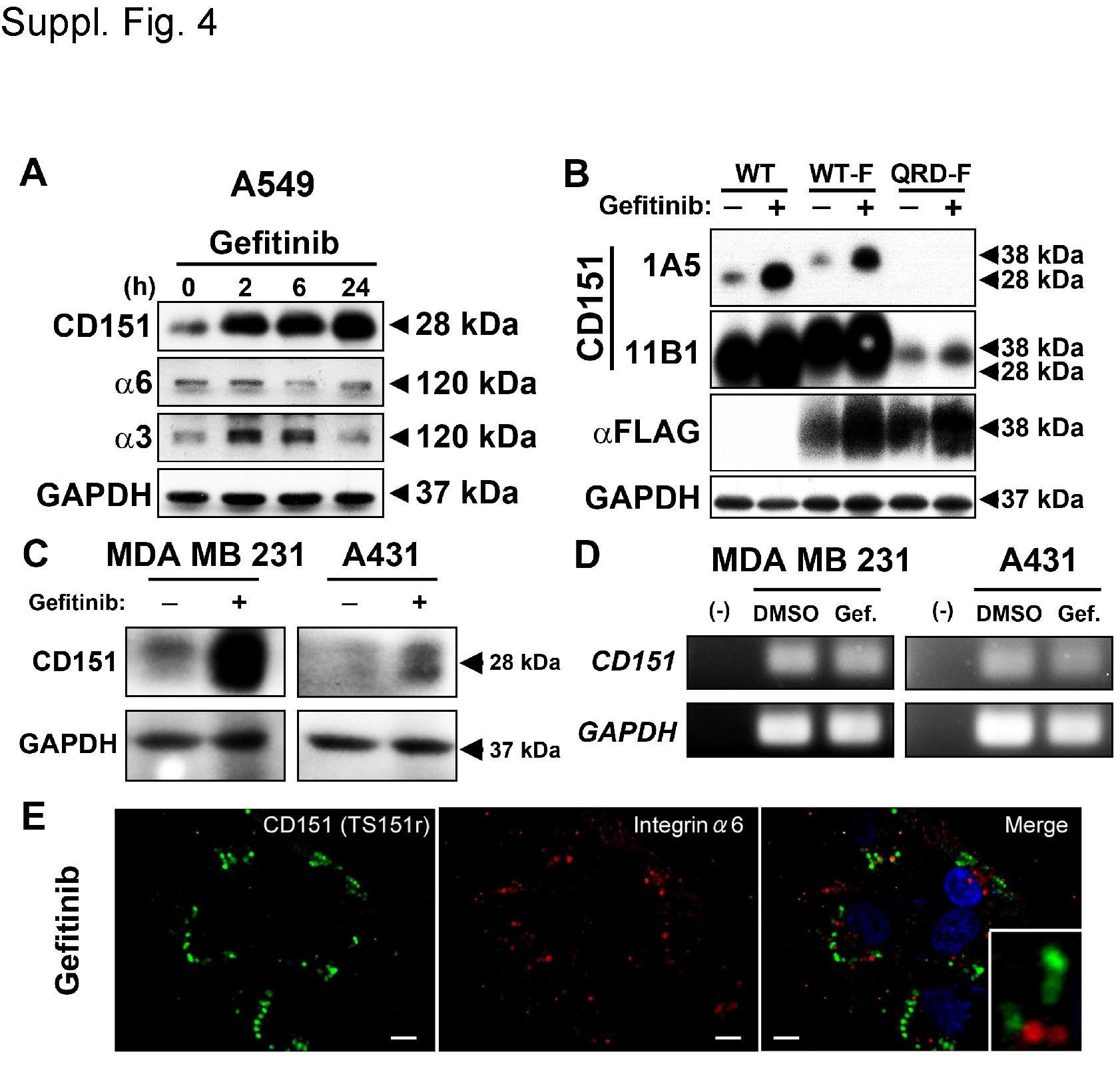

Supplement: Supplementary file 5 — Supplementary material 5 (JPEG 311 kb) [file 18_2019_3014_MOESM5_ESM.jpg]

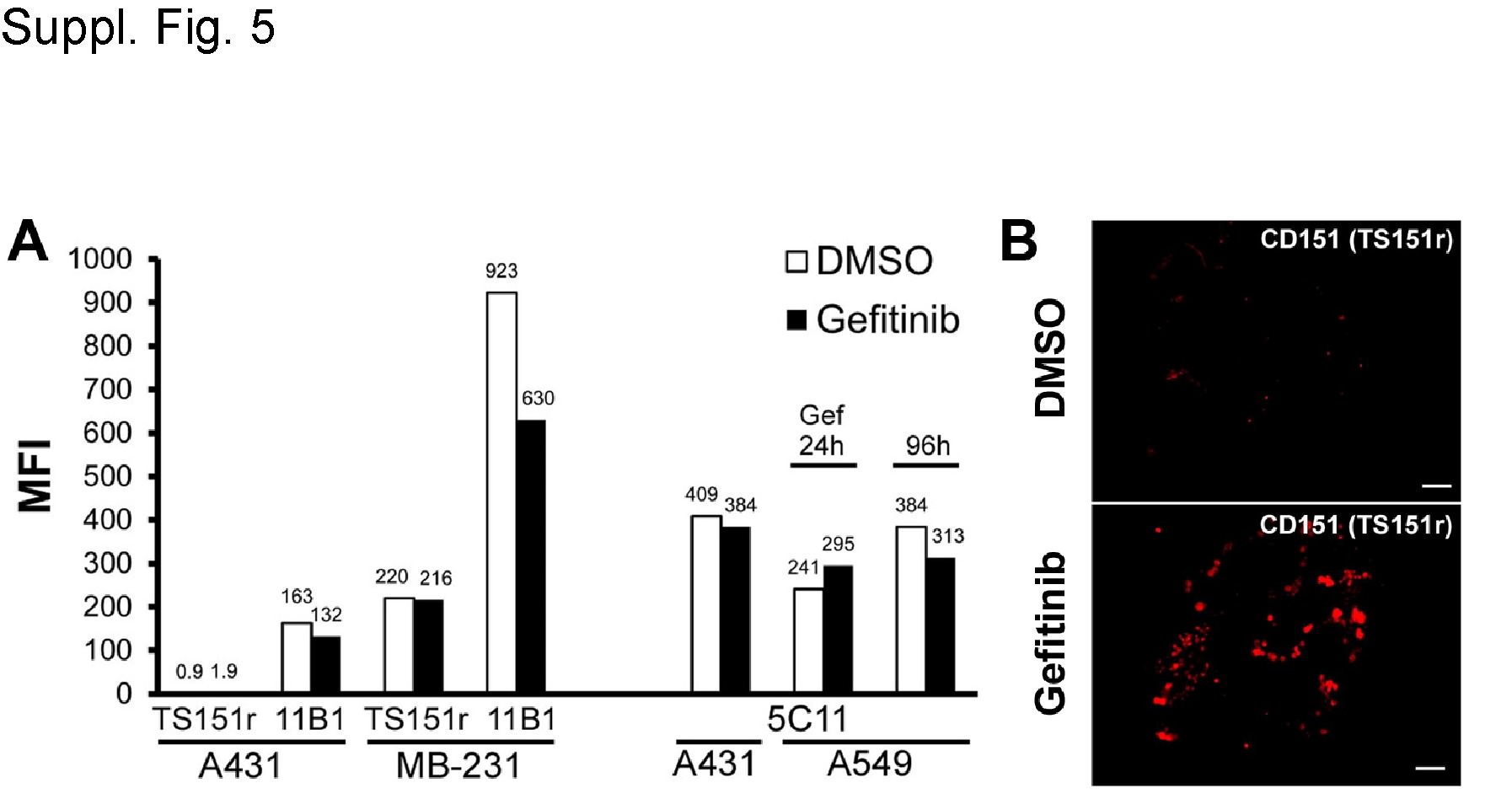

Supplement: Supplementary file 6 — Supplementary material 6 (JPEG 113 kb) [file 18_2019_3014_MOESM6_ESM.jpg]
